# Primary eye care model in India

**Published:** 2022-03-01

**Authors:** Sangeeta Abrol, Kritika Chopra Kumar

**Affiliations:** 1 Professor, Department of Ophthalmology: VMMC & Safdarjung Hospital, New Delhi, India.; 2 Senior Resident Doctor, Department of Ophthalmology: VMMC & Safdarjung Hospital, New Delhi, India.

**The provision of primary eye care within an integrated health care system is a feasible and self-sustaining model of eye care.**

In India, primary eye care forms a critical aspect of primary health care, i.e., eye care is integrated with different multisectoral health schemes, such as the mother and child and non-communicable diseases programmes. Broadly, there are three models of primary eye care prevalent in India—government initiatives, public–private partnerships, and initiatives by non-governmental organisations. This article covers government initiatives.

Eye care services are made available at fixed centres (either standalone or integrated with primary health care centres) or via mobile van service, or through remote consultation.

## National initiatives in eye care

### National programme for control of blindness and visual impairment

The national programme for control of blindness and visual impairment (NPCBVI) was launched in 1976 with the target to reduce the prevalence of blindness from 1.4% to 0.3 % by 2020.^1^ The achievements of the last decade have been encouraging. The rapid survey on avoidable blindness conducted under the programme during 2006–07 showed that the prevalence of blindness reduced from 1.1% in 2001–02 to 1% in 2006–07.^2^

**Figure:**
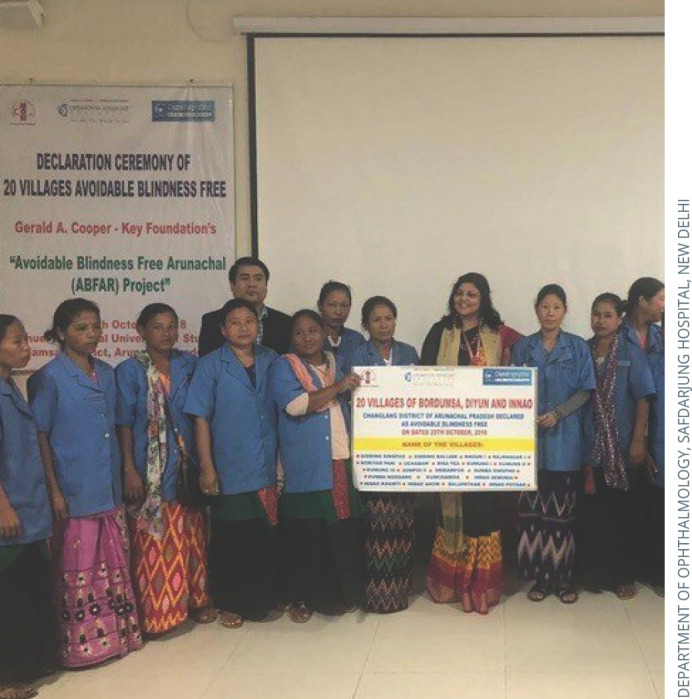
ASHA workers being trained at Changlang district, Arunachal Pradesh. **INDIA**

Since 1994, the school eye screening programme has been an integral part of the NPCBVI. Its initiatives include identifying schools, collecting information on eye care needs of students and teachers, screening and referrals, training of school teachers, and prescribing and providing free spectacles to students from poor socioeconomic backgrounds.^3^

### Ayushman Bharat scheme

**Figure 1:**
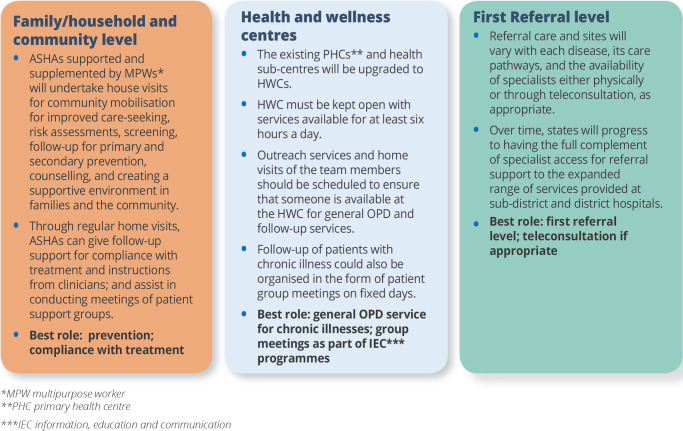
Organization of service delivery under PM-JAY^5^

The Ayushman Bharat scheme, launched in 2018, aims to achieve universal health coverage in India. This is fully funded by the Government of India and has two components:

- Pradhan Mantri Jan Arogya Yojana (PM-JAY): This is a health insurance scheme providing a cover of 0.5 million INR (about USD 6,800) per family for secondary and tertiary care hospitalisation. The beneficiary does not have to pay any user fee or premium under this scheme. The households included are based on the deprivation and occupational criteria of Socio-Economic Caste Census 2011 (SECC 2011).^4^ Importantly, the reimbursement of cataract surgery is an integral part of this programme.
- Creation of health and wellness centres (HWCs): a total of 150,000 existing sub-health centres, primary health centres and urban primary health centres will be transformed into HWCs by 2022, each covering a population of 3,000 to 3,500. Eye care will form an integral component of the comprehensive health care services provided at these centres (Figure 1).^5^ About 70,000 such centres are operational, where 413.5 million people (of which 54% are women) have accessed healthcare. More than 945,000 teleconsultations have been conducted.^6^

## Hub and spoke model of eye care

The provision of primary eye care within an integrated health care system is a feasible and self-sustaining hub and spoke model of eye care. Critical to the success of this model are the following: training existing health care personnel, launching campaigns to inform and educate patients and service providers, and providing essential equipment for screening.

In this context, the role of accredited social health activists (ASHAs) can be strengthened. These health activists are community health workers recruited under the government's National Rural Health Mission.^7^ With appropriate training and sensitisation, ASHAs can play an important role in identifying eye-related problems at the community level and encouraging patients to seek timely primary eye care services locally.

At the heart of the hub and spoke model are vision centres, established at the level of the community health centre. This model is cost-effective, provides comprehensive eye examination, and is a practical means to prevent and control blindness among the underprivileged population. The newer technology of teleophthalmology links those requiring more than primary eye care to secondary and tertiary eye care hospitals.
